# Preparation and Properties Study of a Thermal Conductive Silicone Adhesive Applied in Advanced Packaging

**DOI:** 10.3390/mi17040394

**Published:** 2026-03-25

**Authors:** Yuwen Xu, Liangjun Liu, Wenfei Wang, Minghua Jiang, Haibing Yang, Tingxin Chen, Kun Jia

**Affiliations:** 1 School of Materials Science and Engineering, South China University of Technology, Guangzhou 510641, China; 202411085466@mail.scut.edu.cn; 2 U-Bond Technology Inc., Dongguan 523800, China; liulj@ubondtech.com (L.L.); wangwf@ubondtech.com (W.W.); kevin.jiang@ubondtech.com (M.J.); harry.yang@ubondtech.com (H.Y.); 3 School of Materials Science and Engineering, University of Electronic Science and Technology of China, Chengdu 611731, China; jiakun@uestc.edu.cn

**Keywords:** thermal conductive silicone adhesives, thermal conductivity, shear strength, advanced packaging

## Abstract

In 2.5D/3D stacked advanced packaging, thermal conductive silicone adhesives are widely employed to achieve structural bonding and efficient heat dissipation. In this study, one thermal conductive silicone adhesive was prepared using medium viscosity vinyl silicone oil, hydrogen-containing silicone oil, and micron-sized alumina powder as the primary components. The results demonstrated that the adhesive exhibited excellent thermal and mechanical performance. Specifically, its thermal decomposition temperature exceeded 400 °C, the thermal conductivity reached over 1.80 W·m^−1^·K^−1^, and the thermal resistance was below 12.0 °C·cm^2^·W^−1^. The shear strength exceeded 5.00 MPa. Furthermore, after exposure to an unbiased highly accelerated stress test for 384 h, 1000 thermal cycles, and thermal aging for 1000 h, the adhesive maintained stable thermal conductivity and mechanical properties. The thermal conductivity remained above 1.70 W·m^−1^·K^−1^, and the shear strength remained higher than 5.00 MPa. In addition, the tensile modulus was maintained below 100 MPa, and the coefficient of linear thermal expansion was less than 160 ppm·°C^−1^. Overall, the comprehensive performance of the adhesive satisfies the reliability requirements for advanced packaging substrates and heat dissipation lid assemblies.

## 1. Introduction

In the post-Moore’s Law era, advanced packaging technologies have become increasingly important in semiconductor manufacturing. Driven by the demand for higher performance, greater integration density, and continued miniaturization, chips are evolving at an unprecedented pace, and 2.5D/3D stacked architectures have been widely adopted and commercialized. However, with the proliferation of 2.5D/3D stacked chips, the heat flux generated by densely integrated functional units during operation increases significantly. This results in a rapid rise in the internal temperature of the chip and intensifies thermal stress among micro-scale components [1,2,3,4,5,6,7,8]. It has been reported that for every 2 °C increase in chip temperature, reliability may decrease by approximately 10%. The high heat flux density, therefore, poses a serious threat to device reliability and service lifetime, highlighting the urgent need for efficient thermal management strategies to dissipate excess heat in a timely manner. In chip thermal management, the heat spreader (thermal lid) and thermal interface materials (TIMs) play critical roles [9]. The TIM serves as a filler medium between the heat spreader and the flip chip or substrate, where both high thermal conductivity and strong interfacial bonding are critical. In addition to efficient heat transfer, TIMs must satisfy several requirements, including thermal stability, chemical stability, and mechanical flexibility [10,11]. Throughout the service lifetime of the device, the material must withstand various reliability evaluations while maintaining stable thermal and mechanical performance [12,13,14].

Thermally conductive silicone, typically formulated as a one-part addition-curing system, is a flexible composite consisting of an organosilicone matrix and thermally conductive fillers [15,16,17]. This material exhibits excellent low-temperature resistance, weatherability, electrical insulation, and resistance to moisture and corrosion. Owing to the intrinsic softness of the silicone matrix, it possesses a low elastic modulus and generates minimal internal stress, thereby providing effective stress buffering and vibration-damping capabilities [18,19,20,21]. Although most commercially available one-part addition-curing thermally conductive silicone can achieve thermal conductivity exceeding 1.80 W·m^−1^·K^−1^, their performance may deteriorate under extreme service conditions, such as elevated temperature, high humidity, or other harsh environments [22,23,24,25,26,27]. These adverse service conditions may significantly degrade the mechanical properties of the material, leading to interfacial debonding, cracking, or cohesive failure of the silicone layer. Therefore, in addition to maintaining high thermal conductivity, the material must exhibit adequate mechanical strength, toughness, and long-term stability. Rigorous environmental reliability evaluations enable the early detection of deformation, fatigue, and aging phenomena in thermally conductive silicone, thereby preventing detachment, damage, or failure of the heat spreader assembly. Such reliability assurance is essential to guarantee the thermal management efficiency and long-term operational stability of 3D chip stack assembly and packaging [28,29].

**Figure 1:**
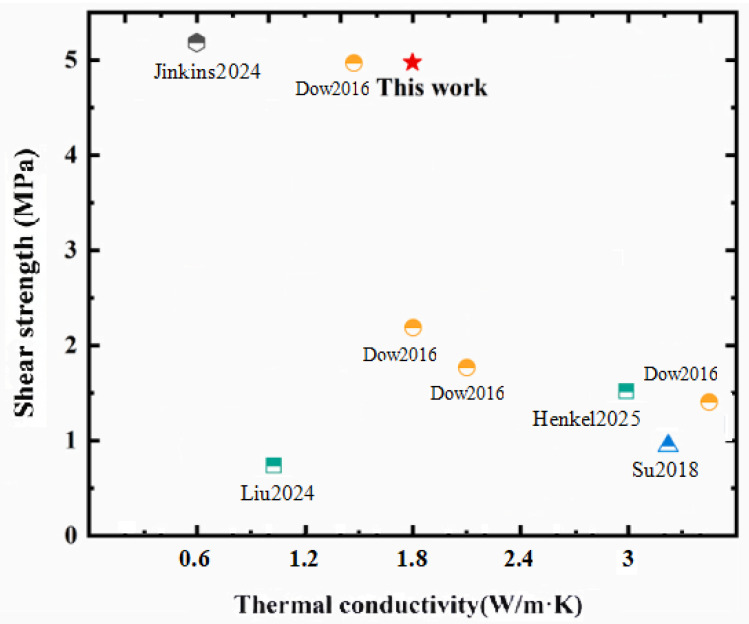
Comparison of thermal conductivity and shear strength of different thermal conductive adhesives [22,30,31,32,33].

As shown in Figure 1, Dow Corning Corporation possesses core technologies for the synthesis of low-modulus silicone materials. However, the single-component thermally conductive silicone adhesive developed by Dow Corning must achieve a balance between thermal conductivity and adhesion strength [30,31]. HENKEL Group similarly has capabilities in organosilicon raw material synthesis, and the thermally conductive silicone adhesive with a thermal conductivity of 3.0 W·m^−1^·K^−1^ has been widely applied in lid-attach bonding for advanced electronic packaging [30]. In this work, a thermally conductive silicone adhesive was prepared by blending vinyl silicone oil, hydrogen-containing silicone oil, composite micron-sized Al_2_O_3_ fillers, catalysts, and agents. Through appropriate formulation design, the resulting silicone adhesive achieved a shear strength of 5.00 MPa and a thermal conductivity of 1.80 W·m^−1^·K^−1^, demonstrating a good balance between adhesion strength and thermal conductivity. The fundamental physicochemical properties of the adhesive were characterized by Fourier transform infrared spectroscopy (FT-IR), differential scanning calorimetry (DSC), thermomechanical analysis (TMA), thermogravimetric analysis (TGA), a universal testing machine, and a thermal conductivity analyzer. In addition, its reliability for advanced packaging applications was evaluated with reference to key reliability tests used in advanced electronic packaging, including the unbiased highly accelerated stress test (uHAST), high-temperature storage test (HTST), and temperature cycling test (TCT). Overall, the obtained thermally conductive adhesive exhibited stable performance and showed potential for applications in advanced electronic packaging.

## 2. Materials and Methods

All reagents used in this study were commercially available. Vinyl-terminated polydimethylsiloxane (Vi-PDMS, Mn ≈ 10,000–30,000 g·mol^−1^) was used as the base polymer. Vi-PDMS was supplied by Zhejiang Runhe High-Tech Materials Co., Ltd. (Huzhou, China). Poly(methylhydrogen)siloxane (PHMS, Mn ≈ 1000–2000 g·mol^−1^) was used as the crosslinker for the hydrosilylation curing system. PHMS was purchased from Shanghai Titan Scientific Co., Ltd. (Shanghai, China). The thermally conductive filler selected was aluminum oxide (Al_2_O_3_) powder. Al_2_O_3_ was widely used as a thermally conductive filler in lid-attach bonding, leveraging its electrical insulation and economic advantages [31,33]. Gamma-glycidoxypropyltrimethoxysilane (KH560) was a conventional coupling agent utilized to enhance interfacial adhesion strength. In this study, KH-560 was employed as the coupling agent in the adhesive formulation. In order to improve the wettability and stability after reliability testing, another additive, polydimethylsiloxane (PDMS, Mn ≈ 300–1000 g·mol^−1^), was incorporated. To accelerate the reaction rate, a platinum catalyst was introduced, specifically an Vinyl siloxane-platinum complex catalyst. Al_2_O_3_ powder and other agents (KH560 and PDMS) were provided by U-BOND Material Technology Co., Ltd. (Dongguan, China). A vinyl siloxane-platinum complex catalyst was obtained from Guangdong Wengjiang Chemical Reagent Co., Ltd. (Shaoguan, China).

**Figure 2:**
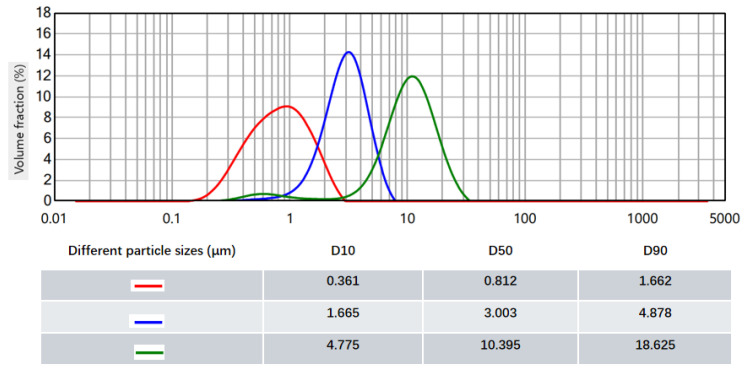
The particle size distribution profiles of the three Al_2_O_3_ powders.

**Figure 3:**
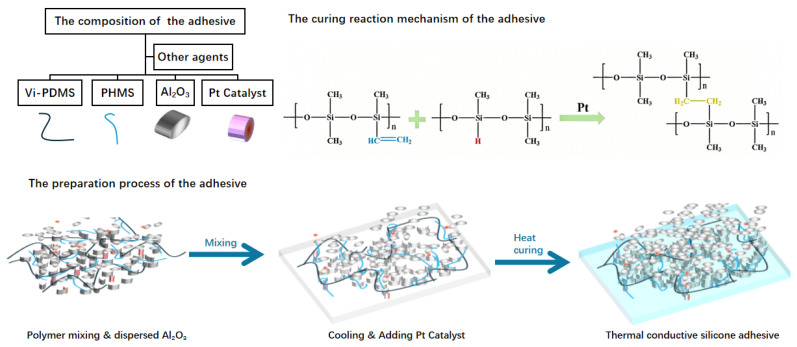
The composition, preparation process and curing reaction mechanism of the thermally conductive silicone adhesive.

To obtain a desirable balance between thermal conductivity and adhesion strength, Al_2_O_3_ fillers were fractionated to achieve different particle sizes and subsequently surface-treated with 0.5–1.0 wt% PDMS. Surface treatment of the fillers was carried out to improve the interfacial wettability between the fillers and the silicone matrix, thereby allowing a higher filler loading. In this work, the thermally conductive adhesive UB-5715 utilized a hybrid filler system consisting of Al_2_O_3_ particles with median diameters (D50) of 0.812 μm, 3.003 μm, and 10.395 μm. Refer to Figure 2 for the particle size distribution profiles of the three Al_2_O_3_ powders. The combination of spherical Al_2_O_3_ particles with three different particle sizes was adopted to enhance filler packing efficiency. This design allows the thermally conductive adhesive to maintain good flowability even at high filler loadings, thereby facilitating the dispensing process [34]. As shown in Figure 3, Vi-PDMS (10–15 wt%) and PHMS (1–3 wt%) were blended with 83–93 wt% of the prepared Al_2_O_3_ powder [35,36]. A DLH-5L power mixer (Foshan Jinyinhe Intelligent Equipment Co., Ltd., Foshan, China) was employed for the preparation process. The mixer features a dual-planetary agitation structure. The impellers execute simultaneous revolution and rotation, generating intense shear forces and kneading effects. This mechanism effectively disaggregates agglomerated powders, ensuring rapid dispersion of the adhesive within a short time frame. The agitation speed was 30–50 revolutions per min at room temperature for 120–180 min to ensure uniform dispersion. The homogeneity of the resulting paste was evaluated microscopically. The dispersion was deemed uniform when the sample exhibited a particle-free morphology without chromatic aberration or heterochromatic spots. After mixing and degassing, the mixture was cooled and then supplemented with 0.1–0.5 wt% KH560, 0.05–0.1 wt% PDMS agents and 0.02–0.05 wt% a vinyl siloxane-platinum complex catalyst. The mixture was further mixed and degassed to obtain the uncured thermally conductive adhesive. Subsequently the adhesive was cured at 125 °C for 2 h using an electric thermostatic blast drying oven (Model DHG-9075A, Shanghai Yiheng Scientific Instruments Co., Ltd., Shanghai, China), which ensured a uniform temperature distribution throughout the chamber. To evaluate its performance at both the material and device levels, the commercially available thermally conductive adhesive DOWSIL™ 4450 (4450, Dow Inc., Midland, MI, USA) was used as a reference sample.

FT-IR spectra were recorded using the attenuated total reflectance method on a Thermo Scientific Nicolet iS5 spectrometer (Thermo Fisher Scientific Inc., Waltham, MA, USA). DSC measurements were carried out on a Netzsch DSC 204 F1 instrument (NETZSCH-Geratebau GmbH, Bavaria, Germany) at a heating rate of 10 °C·min^−1^ under a nitrogen atmosphere. TGA was performed using a Netzsch TG 209 F3 thermal analyzer (NETZSCH-Geratebau GmbH, Bavaria, Germany) at a heating rate of 20 °C·min^−1^. Rheological properties were characterized using an Anton Paar MCR 302e rheometer (Anton Paar GmbH, Graz, Austria). Thermal conductivity and thermal resistance were measured using an LW-9389 thermal resistance tester (Long Win Science & Technology Corp., Taiwan, China). Mechanical properties were evaluated using a CMT6503 universal testing machine (Mettler-Toledo International Inc., Canton of Zurich, Switzerland).

The thermal conductivity and thermal resistance of the conductive adhesive were determined using a steady-state method, based on Fourier’s law, as expressed in Equation (1):

(1) $$k=-\frac{q}{\mathrm{grad}T}=\frac{Q\cdot L}{S\cdot t\cdot \Delta T}=\frac{W\cdot L}{S\cdot \Delta T}$$

where W represents the heating power, L denotes the specimen thickness, S signifies the specimen area and t indicates time [29].

The thermal resistance equation of the specimen at steady-state heat flux is shown in Equation (2):

(2) $$R=\frac{s}{w}\times \Delta T$$

In the formula, R is the thermal resistance, and ΔT is the temperature difference between the hot end and the cold end. Therefore, the relationship between the thermal conductivity (k) and thermal resistance (R) of the specimen can be obtained according to Equations (1) and (2), as shown in Equation (3):

(3) $$R=\frac{1}{k}\cdot L$$

The reliability evaluation was conducted under the following conditions: HTST was conducted in accordance with the JEDEC JESD22-A103 standard [37] under the condition of 150 °C for 1000 h. TCT was performed following the JEDEC JESD22-A104 standard [38], with a temperature range of −40 to 125 °C for 1000 cycles. uHAST per JEDEC JESD22-A118 standard [39] was conducted under high pressure, at 130 °C, and 85% relative humidity, without electrical bias.

## 3. Results and Discussion

### 3.1. Structural Analysis

**Figure 4:**
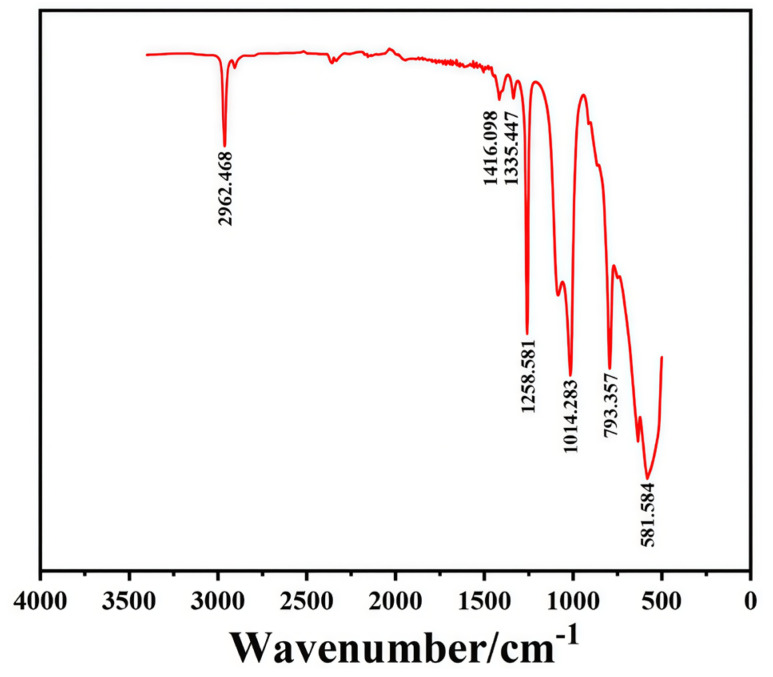
FT-IR spectrum of thermal conductive silicone adhesive UB-5715.

The chemical structure and functional groups of the thermal conductive adhesives were characterized by FTIR, and the resulting spectrum is shown in Figure 4. As observed in the spectrum, a strong absorption peak appeared at approximately 2962 cm^−1^, which is attributed to the asymmetric stretching vibration of the C–H bond in the methyl or methylene groups. The bands located at 1416 cm^−1^ and 1335 cm^−1^ are typically assigned to the bending vibrations of C–H, further confirming the presence of aliphatic chains in the sample. The FTIR spectrum provided clear evidence of the successful formation of the silicone network. Specifically, the absorption band observed at approximately 1258 cm^−1^ is attributed to the asymmetric deformation vibration of the Si–CH_3_ groups, while the prominent peak centered at 1014 cm^−1^ corresponds to the characteristic stretching vibration of the Si–O–Si backbone. Furthermore, the in-plane bending vibration of the –CH_3_ groups was detected near 793 cm^−1^. Crucially, the absence of the characteristic Si–H absorption band (typically located in the 2100–2250 cm^−1^ region) indicates the complete consumption of the PHMS precursor. Collectively, these findings confirm that the hydrosilylation reaction proceeded to completion, resulting in a successfully cross-linked network structure as designed.

### 3.2. Thermal Properties

**Figure 5:**
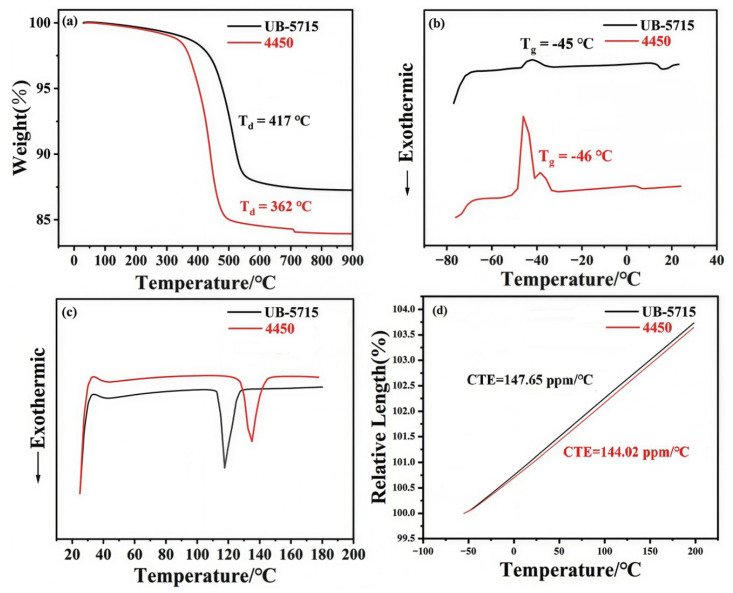
Thermomechanical properties of thermal conductive silicone adhesive (**a**) Thermal weight loss curve at 25~900 °C, (**b**) DSC curve at −75~25 °C, (**c**) DSC curve at 25~180 °C, (**d**) Thermal expansion coefficient curves.

The thermomechanical properties of the conductive silicone were characterized by DSC and TGA for both uncured (wet) and cured (dry) samples, and the corresponding results are presented in Figure 5. UB-5715 exhibited significantly enhanced thermal stability. The decomposition temperature (Td, defined at 2% weight loss) reached 417 °C, which is markedly higher than that of the reference adhesive (362 °C for 4450). These results indicate the superior thermal resistance of UB-5715 under elevated temperature conditions.

The T_g_ of UB-5715 was determined to be −45 °C, which is comparable to that of 4450 (approximately −46 °C). These results indicate that both silicone adhesives remain in a rubbery state at low temperatures, thereby exhibiting excellent low-temperature flexibility and resistance to embrittlement. The exothermic curing peaks of the two thermally conductive silicones were primarily distributed within the temperature ranges of 115–130 °C and 120–150 °C, respectively. To minimize performance variations caused by differences in curing temperature, a unified curing temperature of 125 °C was selected for subsequent sample preparation and testing.

The DSC thermograms of the samples are presented in Figure 5. UB-5715 exhibited a broad exothermic peak centered at approximately 110 °C, corresponding to the crosslinking reaction. In contrast, 4450 displayed two distinct thermal events. The first, a broad exotherm peaking at ~35 °C, indicated a rapid curing reaction occurring at low temperature. The second, a sharp and intense exotherm at ~135 °C, was attributed to the thermal decomposition of the polymer network. These results suggest that while 4450 cures rapidly at ambient conditions, it possesses lower thermal stability compared to UB-5715, which cures at elevated temperatures and forms a more thermally robust network.

To further evaluate the thermal stability and dimensional reliability of cured UB-5715, the coefficient of thermal expansion (CTE) was measured and compared with that of 4450. As shown in Figure 4, the CTE of UB-5715 was 144.02 ppm·°C^−1^, which is 3.63 ppm·°C^−1^ higher than that of the reference adhesive. Despite this slight increase, UB-5715 maintains a relatively low CTE, enabling good dimensional stability over a broad temperature range. Such controlled thermal expansion behavior is beneficial for reducing thermally induced stress in packaging structures and ensuring reliable chip encapsulation under extreme operating conditions.

### 3.3. Rheological Properties

**Figure 6:**
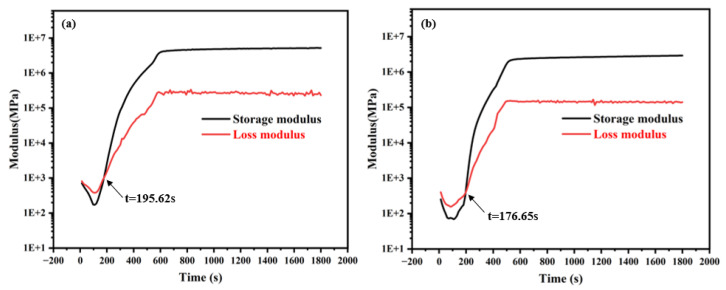
Time dependent variation in storage modulus and loss modulus at 125 °C (**a**) UB-5715, (**b**) 4450.

The curing behavior of the uncured mixtures was investigated using the rheometer under a temperature profile that increased from 25 °C to 125 °C at a heating rate of 5 °C·min^−1^, followed by isothermal holding at 125 °C for 30 min. This analysis aimed to compare the cure kinetics and viscoelastic evolution of UB-5715 and 4450. As depicted in Figure 6, the gelation time of UB-5715 was determined to be 195.62 s, which is notably longer than that of 4450 (176.65 s). This observation aligns well with the DSC thermogram analysis presented in Figure 4. The accelerated reaction kinetics observed in 4450 can be attributed to a preliminary weak exothermic reaction occurring around 35 °C. This early onset of reactivity manifests in the rheological data as a significantly shorter gel time, indicating a more rapid crosslinking process compared to UB-5715.

While the rapid curing of 4450 is advantageous for processing speed, it presents potential risks regarding stress relaxation. Conversely, the slightly extended gel time of UB-5715 allows for a longer low-viscosity phase, facilitating better stress relaxation during the curing process. Consequently, this characteristic results in lower residual cure stress, which is a critical factor contributing to the superior bonding strength exhibited by UB-5715.

### 3.4. Thermal Conductivity

The measured thermal conductivity and corresponding thermal resistance values of UB-5715 and 4450 are summarized in Table 1. In comparison with 4450, UB-5715 exhibits a higher thermal conductivity of 1.80 W·m^−1^·K^−1^, indicating its superior heat transfer capability. This enhanced thermal performance can be attributed to its optimized filler dispersion and improved interfacial thermal pathways within the silicone matrix. Notably, both thermally conductive silicone materials meet the thermal management requirements for flip-chip ball grid array (FCBGA) assembly. Their adequate thermal conductivity ensures efficient heat dissipation during device operation, which is critical for maintaining device reliability, preventing thermal accumulation, and enhancing long-term performance stability in high-power and high-density electronic packaging applications.

### 3.5. The Reliability Evaluation of Thermal Conductive Silicone Adhesive

#### 3.5.1. Reliability of Thermal Conductivity

The measured thermal conductivity and corresponding thermal resistance values of UB-5715 and 4450 are summarized in Table 1. In comparison with 4450, UB-5715 exhibits a higher thermal conductivity of 1.80 W·m^−1^·K^−1^, indicating its superior heat transfer capability. This enhanced thermal performance can be attributed to its optimized filler dispersion and improved interfacial thermal pathways within the silicone matrix. Furthermore, the organic and inorganic additives incorporated within the composite system may gradually undergo decomposition, volatilization, or leaching during extended environmental exposure. These processes can disrupt the continuity and connectivity of the thermally conductive filler network, leading to increased interfacial thermal resistance and diminished heat transfer efficiency.

**Figure 7:**
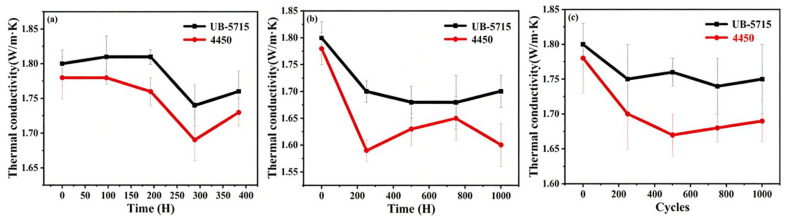
The reliability of thermal conductivity was evaluated under (**a**) uHAST, (**b**) HTST, and (**c**) TCT.

As shown in Figure 7, the reduction in thermal conductivity observed during TCT can be primarily attributed to fatigue effects arising from repeated thermal expansion and contraction. The mismatch in CTE between the silicone matrix and thermally conductive fillers generates cyclic mechanical stresses, which may result in microcrack formation, interfacial debonding, and localized stress concentration. These microstructural defects further hinder the formation of effective heat conduction pathways. Despite these degradation mechanisms, UB-5715 demonstrates remarkable thermal reliability. The maximum reduction in its thermal conductivity during reliability testing is limited to only 5.6% (from 1.80 to 1.70 W·m^−1^·K^−1^), indicating excellent resistance to environmental aging and mechanical fatigue. This superior stability highlights the robustness of its internal filler network and matrix structure, making it highly suitable for advanced electronic packaging applications requiring long-term thermal performance stability.

#### 3.5.2. Reliability of Mechanical Property

**Figure 8:**
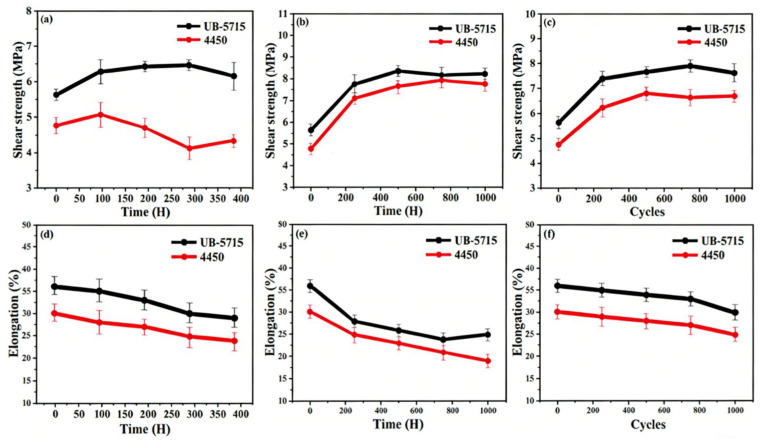
The reliability of shear strength was evaluated under (**a**,**d**) uHAST, (**b**,**e**) HTST, and (**c**,**f**) TCT.

Shear strength is a critical parameter for evaluating the interfacial bonding performance of thermally conductive silicone adhesives. As shown in Figure 8, compared with 4450, UB-5715 exhibited superior shear strength and bonding stability, with values consistently remaining above 5.50 MPa throughout the aging process. Notably, the shear strength of UB-5715 slightly increased after prolonged aging, which may be attributed to further crosslinking or post-curing reactions promoted by elevated temperature and humidity conditions.

In FCBGA packaging, both the stability of the tensile modulus and the coefficient of thermal expansion (CTE) are of paramount importance. A stable and moderate tensile modulus helps maintain structural integrity and stress buffering within the adhesive layer, while a lower CTE reduces thermally induced stress on the chip and interconnects. Moreover, wider temperature excursions can generate greater thermomechanical stress due to CTE mismatch among different packaging components [13].

**Figure 9:**
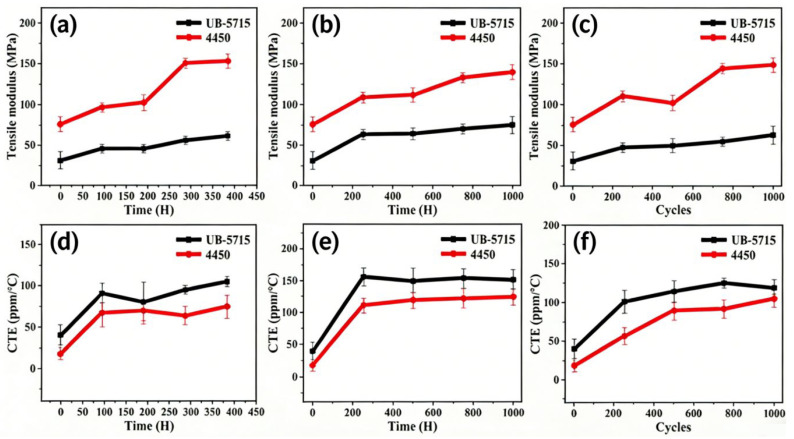
The reliability of tensile modulus was under (**a**) uHAST, (**b**) HTST and (**c**) TCT. And the reliability of CTE was under (**d**) uHAST, (**e**) HTST, (**f**) TCT.

As shown in Figure 9, the average tensile modulus of UB-5715 was lower than that of 4450, remaining below 75 MPa. The relatively lower modulus indicates improved flexibility and stress-buffering capability of the adhesive layer, which is beneficial for maintaining interfacial integrity in packaging applications. The fluctuation amplitude of UB-5715 was approximately 30 MPa, significantly smaller than that of 4450 (approximately 70 MPa), demonstrating superior mechanical stability during aging. Furthermore, the CTE of UB-5715 remained relatively stable throughout the aging process, consistently staying below 160 ppm·°C^−1^. This controlled thermal expansion behavior contributes to reduced thermomechanical stress and enhanced reliability in flip-chip packaging structures.

## 4. Conclusions

In summary, a high thermally conductivity silicone thermal interface material, designated as UB-5715, was successfully prepared using 10–15 wt% of Vi-PDMS and 1–3 wt% of PHMS, combined with 83–93 wt% of micron-sized alumina as the thermally conductive filler. The physicochemical, thermal, and mechanical properties of the material were systematically characterized. The results demonstrate that UB-5715 achieves the targeted thermal conductivity, while exhibiting excellent thermal stability with a Td exceeding 400 °C. The shear strength reached above 5.00 MPa, indicating strong interfacial bonding capability. Furthermore, after exposure to uHAST, HTST, and TCT reliability evaluation, UB-5715 maintained stable thermal conductivity and mechanical performance. Overall, the developed material satisfies the reliability requirements for chip-heat spreader assembly and advanced packaging applications, demonstrating promising potential for practical implementation in high-performance electronic devices.

## Figures and Tables

**Table 1:** Conductive properties of silicone adhesive.

Sample	Thermal Conductivity W·m^−1^·K^−1^	Thermal Resistance °C·cm^2^·W^−1^
UB-5715	1.80	10.947
4450	1.70	11.134

## Data Availability

The original contributions presented in this study are included in the article. Further inquiries can be directed to the corresponding author.
